# Specificity of Cognitive and Behavioral Complaints in Post-Traumatic Stress Disorder and Mild Traumatic Brain Injury

**DOI:** 10.3390/bs5010043

**Published:** 2015-01-30

**Authors:** Hélène Pineau, André Marchand, Stéphane Guay

**Affiliations:** 1 Department of Psychology, Université du Québec à Montréal, Montreal, QC H3C 3P8, Canada; E-Mail: hpino@hotmail.com; 2 Lucie-Bruneau Rehabilitation Centre, Montreal, QC H2H 1C5, Canada; 3 Institut Universitaire en Santé Mental de Montréal, Montreal, QC H4H 1R3, Canada; E-Mail: stephane.guay@umontreal.ca; 4 Department of Criminology, Université de Montréal, Montreal, QC H3C 3P8, Canada

**Keywords:** cognitive and behavioral complaints, PTSD, MTBI, comorbidity, depression, anxiety

## Abstract

Characterization of cognitive and behavioral complaints is explored in Post-traumatic stress disorder (PTSD) and mild traumatic brain injury (MTBI) samples according to the severity of PTSD, depression and general anxiety conditions. Self-reported questionnaires on cognitive and behavioral changes are administered to PTSD, MTBI, MTBI/PTSD and control groups. Confounding variables are controlled. All groups report more complaints since the traumatic event. PTSD and MTBI/PTSD groups report more anxiety symptoms, depression and complaints compared to the MTBI group. Relatives of the PTSD group confirm most of the behavioral changes reported. Results suggest the utility of self-reported questionnaires to personalize cognitive and behavioral interventions in PTSD and MTBI to cope with the impacts of the traumatic event.

## 1. Introduction

Clinical psychologists who work with individuals with post-traumatic stress disorder (PTSD) are often faced with complaints of persistent cognitive problems, including difficulties with memory and concentration, which can cause a decrease in the effectiveness of the therapeutic interventions. It frequently happens that such cognitive or behavioral changes may be ignored or minimized during the initial hospitalization when severe psychological reactions or obvious physical injuries are present [1].

Mild traumatic brain injuries (MTBI) represent approximately 85% of all traumatic brain injuries in North America [2]. The cluster of acute symptoms that develop following an MTBI is referred to as post-concussion syndrome (PCS). The symptoms (e.g., headaches, dizziness, irritability, memory and concentration problems) become chronic in 5% to 15% of individuals [3] and tend to partially overlap or be confounded with PTSD symptoms [4]. PTSD following a traumatic event further complicates the evaluation, the diagnosis and the treatment of the physical and neurological injuries.

The PTSD clinical population may also present with MTBI subsequent to an accident, physical assault or military operation. For example, MTBI is the second most common problem among injured survivors of the military operations in Iraq and Afghanistan, second only to orthopedic injuries, according to Tanelian and Jaycox [5]. Not surprisingly, the literature in this area refers to MTBI as the “signature injury” in these two military conflicts. MTBI most commonly occurs during attacks by improvised explosive devices (IED). Survivors may present concomitant PTSD subsequent to the same event, thereby further complicating the evaluation, diagnosis and treatment of the physical and neurological injuries. Neuropsychologists may wish to refer such patients to psychologists specialized in PTSD to determine whether or not psychological treatment is recommended. A client may present persistent PCS that may have been hidden or exacerbated by the psychological condition.

In the context of medical or psychological healthcare services for individuals with PTSD and/or MTBI, psychologists and neuropsychologists rarely work in an interdisciplinary environment that would allow the two problems to be simultaneously addressed through, for example, a combined treatment plan. Given the lack of opportunity for interdisciplinary treatment, the use of questionnaires measuring the nature and the intensity of cognitive and behavioral changes post-event should be considered. Such questionnaires are fast, cost-effective and allow clinicians to improve the planning and the orientation of their evaluations and their treatment.

The few studies that address cognitive problems in MTBI and PTSD primarily focus on military veterans with PTSD [6] or on civilians with MTBI [7,8]. Two studies [9,10] explored the nature of complaints in individuals with PTSD and self-reported MTBI. Hoge and McGurk [10] noted that the characteristics of the physical and cognitive complaints of MTBI (e.g., brief loss or alteration in consciousness) reported by veterans from Iraq are also characteristic of the PTSD and comorbid depression symptoms. The authors inferred a mediating role of PTSD and depression in the expression of physical and cognitive symptoms in veterans with presumed double diagnoses. Vanderploeg and Belanger [9] studied a mixed group comprising soldiers and civilians with chronic PTSD and subsequent MTBI sustained several months or years later. Their results suggested that when MTBI occurs in the context of pre-existing PTSD, the effectiveness of psychological treatment for the traumatic symptoms is compromised. According to Vanderploeg and Belanger [9], PTSD contributes more significantly to subjective physical, cognitive and emotional symptoms than MTBI and that neither condition moderates the symptomatology of the other; rather, their effect is cumulative.

Certain methodological problems in previous studies limit the generalization of the findings. For example, the conclusions of two previous studies [9,10] are respectively based on statistical analyses conducted with data from a few general questions concerning attention and memory and from clinical populations whose diagnoses were established from subjective, retrospective reports. Furthermore, the MTBI and PTSD diagnoses were not based on formal medical or psychological evaluations, and the absence of a control group precludes information about baseline symptoms in the general population. Finally, some study designs did not adequately control for the presence of pre-existing or concurrent medical, neurological and developmental conditions, making it difficult to know if participants’ reports of their complaints were unaffected by these conditions.

The objective of the present study was to describe and document the subjective cognitive and behavioral symptoms of PTSD and MTBI, while considering some of the methodological problems of past studies. In light of the exploratory nature of the study and the limited existing literature in the area, three research questions were developed: (1) Given the overlap in clinical symptomatology between PTSD and MTBI, are the symptoms reported by individuals in these two groups similar in nature and intensity? (2) Does the presence of comorbid MTBI and PTSD intensify the expression of symptoms in a “cumulative” effect, as suggested by Vanderploeg and colleagues (2009)? (3) Are the complaints reported by the three clinical groups independently validated by relatives, as in Biddle and colleagues (2002)?

## 2. Methods

### 2.1. Participants

In total, 75 subjects were distributed into four groups in the study. The subsample sizes are specified in each of the following subsections. Reports of cognitive and behavioral symptoms were gathered from three groups of participants with psychological and/or neurological trauma: individuals with PTSD, individuals with MTBI and individuals with a double diagnosis of PTSD and MTBI.

#### 2.1.1. PTSD Group

Twenty five civilian participants with a formal diagnosis of PTSD were included in the study. The participants were recruited through advertisements in the community or were referred by medical or mental health professionals at the Centre d’Étude sur le Trauma (Trauma Studies Center) at the Institut Universitaire en Santé Mentale de Montréal.

#### 2.1.2. MTBI Group

Nineteen civilian participants with a formal diagnosis of MTBI (based on medical records of the accident or assault) were included in the study. Criteria for MTBI were based on the international definition of mild traumatic brain injury (see Carroll and Cassidy [3]). The criteria were as follows: period of altered consciousness for less than 30 min, Glasgow Coma Scale score between 13 and 15 and post-traumatic amnesia (PTA) for less than 24 h. Formal MTBI diagnoses were established by a doctor (neurologist or emergency physician) following admission to the emergency department or later, during the medical evaluation process of neurological sequelae. Participants were recruited in a tertiary rehabilitation centre for traumatic brain injury (Centre de Réadaptation Lucie-Bruneau (Lucie Bruneau Rehabilitation Centre)) in Montreal.

#### 2.1.3. MTBI/PTSD Group

Following the evaluation of the medical history and the psychiatric condition, six participants with MTBI were also diagnosed with PTSD (MTBI/PTSD) and were recruited from the Lucie Bruneau Rehabilitation Centre following evaluation using the SCID-I (the Structured Clinical Interview for DSM-IV for Axis I).

#### 2.1.4. Control Group

The twenty-five participants in this group were recruited through advertisements in the newspapers or on the Internet. Each participant in this group was between 18 and 60 years old and was recruited to be as close in age and gender to both PTSD and MTBI groups as possible.

### 2.2. Language and Litigation

The PTSD and MTBI groups matched in ethnicity and language (mostly Caucasian, with French as their first language). The few foreign participants in each group were screened over the telephone to ensure that their oral and written French were adequate. Nine participants in the PTSD group were involved in litigation, and one was excluded from the study, as the subject did not meet the symptoms criteria during the evaluation process. None of the participants in the MTBI group were involved in litigation at the time of the evaluation.

### 2.3. Exclusion Criteria

Exclusion criteria for the present study were the following: (1) unstable medical conditions, past history of traumatic brain injury before the more recent one or other diseases with the potential to affect brain functioning in pre- or post-trauma; (2) existing substance abuse problem; (3) learning disorder or attention deficit in school; (4) history of physical violence during infancy; (5) an incapacitating physical disorder that is not adequately controlled; (6) a psychotic episode (past or present); and (7) a bipolar disorder or an organic mental disorder.

Of the 135 participants recruited or referred to the study, we excluded thirty five PTSD participants, twenty two MTBI participants and two controls. The percentage of excluded participants in each group was as follows: 58.3% in the PTSD group (35 excluded/60 recruited), 52.4% in the MTBI group (22/42), 7.4% in the control group (2/27) and 0 in the MTBI/PTSD group (0/6). The primary reasons for exclusion, in descending order, were the following: dropped out or were no longer interested (11 PTSD; 5 MTBI); suspected or confirmed prior brain injury (9 PTSD; 1 control); uncontrolled medical condition (6 PTSD; 3 MTBI; 1 control); age over 60 years (3 PTSD; 4 MTBI); history of drug or alcohol abuse (2 PTSD; 4 MTBI); insufficient French language skills (2 PTSD; 2 MTBI); suspected visual, sensory or motor problems (2 MTBI); developmental attention problems (1 PTSD; 1 MTBI); suspected bipolar disorder (1 MTBI); and suspected malingering (1 PTSD). The fact that 50% of the recruited participants in the PTSD and MTBI groups were excluded confirms the importance of controlling for the high comorbidity that can occur in these populations to avoid attributing participants’ complaints to conditions other than PTSD and MTBI.

### 2.4. Procedure

Subjects were informed that participation in the study was voluntary. All participants provided written consent prior to the study. As a prerequisite for entering the study, participants underwent a complete psychological screening, including a formal evaluation to establish the presence of PTSD as a primary condition and to evaluate possible secondary conditions, such as anxiety. Self-report questionnaires regarding cognitive and behavioral changes observed post-trauma were completed in the context of a neuropsychological evaluation (results not presented here).

The study was conducted at Louis-H Lafontaine Hospital in Montreal (Quebec, Canada) and at the Lucie Bruneau Rehabilitation Centre. The study was approved in January 2006 (#CER CRIR-138-0405), by the ethics committees at Louis-H Lafontaine Hospital in Montreal (Quebec) and the Centre de Recherche Interdisciplinaire en Réadaptation (CRIR) (Centre for Interdisciplinary Research in Rehabilitation), with which the Centre de Réadaptation Lucie-Bruneau was affiliated. The same evaluator met with each participant at one or the other centre, according to the origin of the referral. Questionnaires were completed at home between evaluation sessions.

### 2.5. Assessment

#### 2.5.1. Psychological Condition

Participants were included in the PTSD group only if PTSD was the primary diagnosis and if all other psychological conditions (e.g., depression) were secondary to PTSD. The diagnoses were established using the Structured Clinical Interview for DSM-IV for Axis I disorders (SCID; Firsta nd Spitzer [11]). The Clinician Administered PTSD Scale for DSM-IV-Revised (CAPS; Blake, Weathers [12]) was administered to establish the frequency and severity of PTSD symptoms in the PTSD and MTBI groups. A trained psychologist, who was blind to the participants’ psychological conditions, administered the structured interviews. The Beck Depression Inventory (Beck-II; Beck, Steer [13]) was used to evaluate symptoms of depression, while the intensity of anxiety symptoms was measured with the State-Trait Anxiety Inventory (STAI; Spielberger, Gorsuch [14]). Although participants taking medication (antidepressants, anxiolytics and pain killers) were not excluded, the medication regimens had to remain stable during the evaluation process. We screened for eligible participants until achieving the intended sample size, and we excluded those presenting the exclusion criteria described before.

#### 2.5.2. Assessment of Cognitive and Behavioral Complaints

The Cognitive Failure Questionnaire (CFQ; Broadbent, Cooper [15]) and the Frontal Systems Behavior Scale (FrSBe; Grace and Malloy [16]) were used to evaluate cognitive and behavioral problems. The CFQ is a 25-item self-report measure of cognitive failure in perception (e.g., failure to find an item at the supermarket, although it is there), memory (e.g., failure to remember names) and motor function (e.g., inadvertently bumping into people) in everyday functioning. The five-point Likert scale measures frequency, from 0 (never) to 4 (very often), for each cognitive failure. For the purpose of the present study, we identified two time periods (pre- and post-trauma/accident) for the PTSD group.

The Frontal Systems Behavior Scale [16] measures behavior associated with frontal lobe dysfunction. It is comprised of 46 items divided in three subscales: apathy, disinhibition and executive yield three subscales scores and a total score. In addition to the self-report form, the scale includes a rating form to be completed by a relative as a way to provide independent assessments of the behavior problems reported by the participant. Participants and relatives used a five-point Likert scale to estimate the frequency of each behavior from 1 (never) to 5 (almost always) pre-/post-event. The pre- and post-conditions are defined by the subject’s perception “before the traumatic event” (pre-) and “actual” (post-), *i.e.*, since the traumatic event. For the control group, the subjects only reported their perception for the actual condition. Relatives completed the questionnaire and returned it by mail, anonymously. All of the questionnaires used in the present study have good psychometric properties.

#### 2.5.3. Statistical Analyses

**Figure 1:**
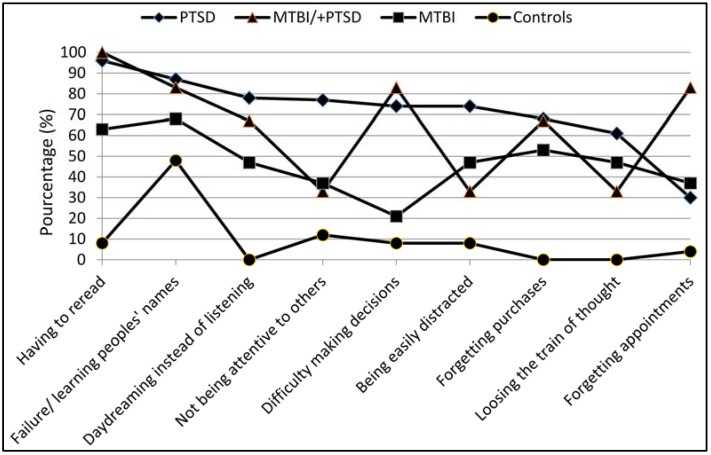
Example of most frequent responses to Cognitive Failure Questionnaire (CFQ) (“often” and “very often”).

**Figure 2:**
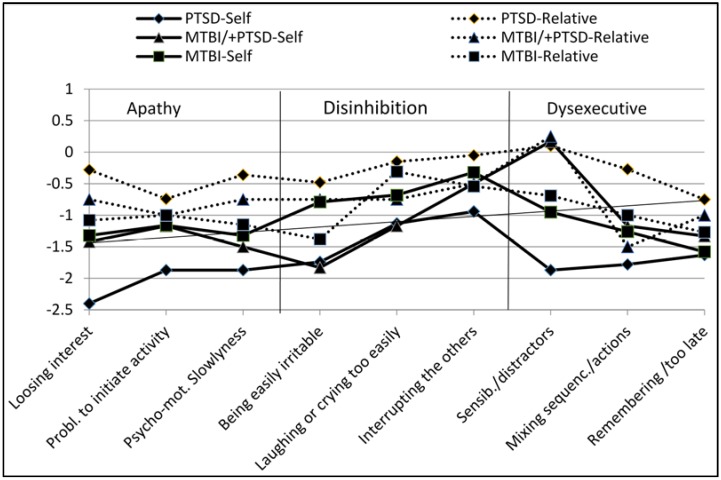
The most common changes reported by clinical groups on FrSBe dimensions.

The first step in the statistical procedure was to compare the results of the three clinical groups with those of the control group for age, gender, education and elapsed time since the trauma, in order to determine whether or not the groups were equivalent (see Table 1). Second, the clinical and control groups were compared on clinical variables, including depression (BDI-II) and trait and state anxiety (STAI). Third, the results were obtained by the clinical and control groups on the measures of cognitive (Figure 1) and behavioral problems (Table 2 and Figure 2), to determine whether or not the clinical groups’ symptoms were greater than the symptoms reported in the general population. Finally, multiple regression analyses were conducted to establish the relative independent contributions of depressive, anxious and post-traumatic conditions to the intensity of reported cognitive problems, behavioral problems and fatigue.

**Table 1:** Summary of clinical and control variables.

	PTSD	MTBI	MTBI/PTSD	Controls	*F (df)*	Part. η^2^
*Variables*	(*n* = 25)	(*n* = 19)	(*n* = 6)	(*n* = 25)		
*Control*	*M (SD)*	*M (SD)*	*M (SD)*	*M (SD)*		
Age	38.5 (12.4)	40.3 (14.7)	33.3 (15.9)	38.9 (12.6)		
Female ^a^ Education	19/25 (76.0%) 14.8 (3.1)	9/19 (47.4%) 13.4 (3.9)	2/6 (33.3%) 12.8 (3.1)	19/25 (76.0%) 15.4 (2.6)		
Elapsed time (in months)	48.5 (41.8) (range 2–146)	30.7 (31.6) (3–98)	9.0 (2.9) (4–11)	N/A	*F* (2, 47) 3.44 *	0.13
*Clinical*	*M (SD)*	*M (SD)*	*M (SD)*	*M (SD)*		
BDI-II Total score	29.8 ^b^ (13.0)	11.6 ^d^ (11.4)	23.8 ^b^ (10.6)	4.8 ^b^ (5.0)	*F* (3, 65) 25.81 **	0.54
STAI State	54.0 ^c^ (11.0)	37.6 ^c^ (12.9)	43.3 (7.8)	29.3 (6.3)	*F* (3, 67) 25.65 **	0.53
STAI Trait	59.5 ^c^ (10.2)	41.1 ^c^ (14.3)	55.8 (8.6)	32.6 (10.4)	*F* (3, 67) 25.18 **	0.53

Notes: BDI-II = Beck Depression inventory version II; STAI State = State-Trait Anxiety Inventory, state version; STAI Trait = State-Trait Anxiety Inventory, trait version. ^a^ The female/male ratio is not equivalent across groups (Fisher’s exact test, *p* = 0.047). Due to missing data, *n* was 1 ^b^, 2 ^c^ or 3 ^d^ lower. * *p* < 0.05. ** *p* < 0.01.

**Table 2:** Participants’ self and relatives’ Frontal Systems Behavior Scale (FrSbe) scores.

Means and Standard Deviations for Behavioural Dimensions Post-Event						
Group ^a^	PTSD	MTBI	MTBI/PTSD	Control		
Dimension	*M* (*SD*)	*M* (*SD*)	*M* (*SD*)	*M* (*SD*)	*F (df)* ^b^	Part. η*^2^*
*Apathy*						
Self	43.3 (9.7)	33.8 (10.0)	43.3 (11.0)	23.6 (6.4)	21.96 **	0.49
Relative	36.7 (10.6)	30.1 (12.7)	42.2 (5.4)	23.9 (6.7)	7.59 **	0.30
*Disinhibition*						
Self	36.5 (8.5)	32.1 (9.6)	37.8 (7.1)	25.8 (5.1)	9.12 **	0.28
Relative	31.8 (7.3)	30.5 (11.6)	38.8 (9.6)	25.7 (6.5)	3.67 *	0.17
*Executive*						
Self	48.0 (9.6)	42.9 (13.2)	50.1 (13.7)	28.8 (6.6)	17.23 *	0.43
Relative	46.2 (11.1)	41.3 (17.2)	53.2 (10.2)	31.9 (11.5)	5.66 **	0.25
*Total*						
Self	127.8 (23.7)	108.8 (28.1)	131.2 (29.8)	76.6 (18.9)	21.26 **	0.48
Relative	114.7 (26.1)	102.0 (39.5)	134.2 (18.2)	79.3 (18.0)	7.97 **	0.31

Note: Only post-trauma scores are reported. There were no significant group differences for pre-event scores. ^a^ Due to missing data, *n* differs for self and relative scores, respectively, in the PTSD group (*n* = 23 and 20), the MTBI group (*n* = 19 and 13), the MTBI/PTSD group (*n* = 6 and 5) and the control group (*n* = 25 and 18). ^b^ For self-scores, *df* = (3, 69), and for relative scores, *df* = (3, 52). * *p* < 0.05. ** *p* < 0.01.

Descriptive and parametric analyses were conducted using SPSS version 15. Fisher’s exact test was used to compare the male-to-female ratios between groups. One-way ANOVAs were conducted on four levels of dependent variables (groups). *Post hoc* analyses compared two groups at the same time by means of a *t*-test. Statistical significance was set at *p* < 0.05. When the distribution of pooled data scores did not respect the assumptions of normality, score transformations (square roots or logarithms) were performed. Questionnaires with over 10% missing data were excluded from analyses. Missing data were replaced using the mean substitution method.

## 3. Results

### 3.1. Control and Clinical Variables

Significant differences in means on control and clinical variables are reported in Table 1.

There were no statistically significant group differences in age or education level, but significant differences between groups (Fisher’s exact test, *p* < 0.05) were observed in the ratio of male to female participants. The MTBI/PTSD group reported significantly less elapsed time since the trauma than did the PTSD group. Regarding clinical variables, a significant main effect of group was found on the BDI and STAI inventories. *Post hoc* analysis revealed that the PTSD and MTBI/PTSD groups were significantly more depressed than the control (*t*(1, 48) = 8.97, *p* < 0.05 and *t*(1, 29) = 6.60, *p* < 0.05, respectively) and MTBI group (*t*(1, 42) = 4.85, *p* < 0.05 and *t*(1, 23) = 2.32, *p* < 0.05, respectively). For anxiety dimensions, the PTSD group’s scores were significantly different from control (PTSD group for anxiety state: *t*(1, 48) = 9.74, *p* < 0.05; and for anxiety trait: *t*(1, 48) = 9.23, *p* < 0.05, respectively; MTBI/PTSD group for anxiety state: *t*(1, 29) = 4.68, *p* < 0.05; and for anxiety trait: *t*(1, 29) = 5.05, *p* < 0.05, respectively). Finally, the MTBI group did not differ significantly from controls on depression and anxiety scores (all *post hoc* comparisons >0.05).

### 3.2. Primary and Secondary Diagnoses

Table 3 qualitatively resumes the distribution of the severity of PTSD conditions. This severity of the PTSD condition was established by clinicians based on the SCID interview. The data only concern the PTSD and MTBI/PTSD groups, since MTBI and control, by definition, do not have PTSD according to the inclusion criteria. The proportion of participants suffering from a PTSD diagnosis specified as moderate and severe (as assessed by the SCID-I) was greater in the PTSD group (64%) than in the MTBI/PTSD group (17%). Furthermore, 32% of participants in the PTSD group reported severe PTSD diagnosis, while none did in the MTBI/PTSD group. In the latter group, the majority of participants presented mild and moderate PTSD diagnoses (33%). Furthermore, participants presented sub-clinical symptoms (33%) or partial PTSD (17%) in the MTBI group. In contrast, only 4% of participants in the PTSD group presented sub-clinical symptoms at the time of the evaluation. Based on Mylle and Maes’ [17] recommendations, the “sub-clinical” PTSD category includes the cases that did not reach the number of symptoms required for Criterion C (avoidance) or D (neurovegetative hyperactivity), although at least one symptom of every criterion was present. The second category indicated by the term “partial” PTSD refers to the cases where one or another of the criteria is missing (intrusion and hyper-awakening) in spite of the significant presence of the F criterion.

Results on the SCID revealed that the PTSD group presented more secondary diagnoses than did the MTBI group. Secondary diagnoses reported by participants in the PTSD group included mood and anxiety disorders.

**Table 3:** Percentage of the PTSD group according to the severity of symptoms.

Severity of Symptoms	PTSD (%)	MTBI/PTSD (%)
Severe	32	0
Moderate/severe	64	33
Light/moderate	0	17
Partial/sub-clinical	4	50

### 3.3. Cognitive and Behavioral Symptoms

#### 3.3.1. Cognitive Failure Questionnaire

Higher scores on the CFQ indicate more reported cognitive failures. Significant main effects of group were observed pre-/post-traumatic event, *F*(3, 69) = 3.48, *p* = 0.02 and *F*(3, 69) = 20.03, *p* = 0.00, respectively. *Post hoc* comparisons confirmed a statistically significant difference post-event between the control (*M* = 28.60, *SD* = 11.93) and clinical groups (PTSD: *M* = 63.20, *SD* = 13.33; MTBI: *M* = 48.95, *SD* = 23.37; MTBI/PTSD: *M* = 58.50, *SD* = 9.83). Comparisons between clinical groups revealed that participants in the PTSD group reported significantly greater distractibility (*p* < 0.05) than did the MTBI participants. Figure 1 shows the percentage of individuals in each group who endorsed the most common complaints (“often” and “very often” combined) since the event. Across the nine most frequently reported complaints, the median percentages were 57%, 37%, 50% and 8% for the PTSD group, MTBI group, MTBI/PTSD group and control group, respectively.

As illustrated in Figure 1, more than 70% of participants in the PTSD group reported being easily distracted and not attentive to others, while 83% of participants in the MTBI/PTSD group reported forgetting appointments. Needing to reread material due to difficulty concentrating was reported by 95% of participants in the PTSD and MTBI/PTSD group, but only 63% of the MTBI group.

#### 3.3.2. Self-Report of Frontal Behavior (FrSBe)

Table 2 describes the mean self-report and relative report scores on the FrSBe scale for the participant’s behavior since the event. Higher scores indicate greater perceived behavior changes. The total score is the sum of three dimensions of frontal behavior: apathy, disinhibition and executive dysfunction. ANOVA tests were conducted between the pre- post-event scores of all groups (except for the control group, since this group has, of course, no post-event condition). The main effects are presented first, followed by the participants’ most frequently reported qualitative changes since the event.

Significant main effects were found between all groups for the total and subscale scores. *Post hoc* comparisons confirmed that the clinical groups reported significantly more complaints than controls (pre-event), except for the MTBI group, which did not differ from the control group on disinhibition dimension (*p* = 0.07). PTSD participants self-reported significantly more apathetic behavior (*t* (1,42) = 3.18, *p* < 0.05) since the event (mean self-score = 43.3) than the MTBI group (mean self-score = 33.8). No other comparisons between clinical groups reached statistical significance.

#### 3.3.3. Perceptions of Relatives

A significant main effect of group was found for every dimension, as well as for total score, on the FrSBe family version (see Table 2). *Post hoc* comparisons confirmed that the mean scores for the PTSD and MTBI/PTSD groups’ relative reports for apathy and executive dysfunction were significantly different from the means of the control group’s relative reports on these dimensions. Only relatives of participants in the MTBI/PTSD reported significantly more complaints on the disinhibition dimension post-event. Despite complaints reported by the MTBI group, their relatives did not report significant changes on any of the FrSBe dimensions since the event. The MTBI/PTSD and PTSD groups reported more problems since the event on all three FrSBe subscales, but only the former group’s complaints since the trauma were externally validated by relatives.

#### 3.3.4. Most Frequently Reported Behavioral Changes

Figure 2 shows the most frequent changes reported by clinical groups and their relatives for each behavioral dimension post-trauma. The scores represent the difference in pre-/post-event scores for the most frequently reported items in each group. Negative scores indicate change (in the sense of deterioration) in behavior; a score of zero indicates no perceived change post-event.

As shown in Figure 2, the participants in the PTSD group reported the most perceived change post-event. The biggest discrepancies between participants and their relatives in the perceived most frequent changes were in the PTSD group.

## 4. Discussion

The present exploratory study was designed to respond to three research questions: (1) Given the overlap in clinical symptoms between PTSD and MTBI, are the cognitive and behavioral problems reported by individuals in the two groups similar in nature and intensity? (2) Does the presence of comorbid MTBI and PTSD increase the intensity of symptoms? (3) Are the symptoms reported by the three clinical groups (MTBI, PTSD and MTBI/PTSD) independently validated by close relatives?

### 4.1. Differences in Intensity and Specificity of Cognitive and Behavioral Complaints in PTSD and MTBI Groups (Question 1)

In the present study, the three clinical groups reported greater cognitive problems since the traumatic event than did the control group. The PTSD group reported significantly more daily distractibility and forgetfulness than did the MTBI group, while the MTBI group reported significantly more distractibility than did the control group. The qualitative analysis of individual symptom profiles allowed us to identify the symptoms most frequently reported by the participants in each group or by their relatives.

The results on behavioral change revealed that the three clinical groups reported more apathetic, disinhibited and disorganized behavior than did the control group. Only the MTBI group does not differ from the control on the self-reported measure of disinhibition. The PTSD group reported significantly greater apathy post-event than did the MTBI group. Irritability figured among the most common qualitative changes reported by the PTSD group, but not by the MTBI group. The predominant manifestation of apathy reported by participants in the PTSD group was loss of interest in initiating and participating in activities; this complaint was not common among participants in the MTBI group. As for executive functioning, a significant increase in distractibility was reported by participants of the PTSD group, but not as much by participants in the double diagnosis or MTBI groups.

In sum, the findings suggest that the PTSD group reported more complaints than the MTBI group and that some complaints could differ qualitatively between groups. However, these qualitative observations must be replicated in more subjects to ensure the significance of the present qualitative observations. However, the specific contribution of post-traumatic stress to this result is difficult to establish due to the strong comorbidity of anxious and depressive symptoms associated with trauma symptoms in individuals with PTSD (with or without MTBI). Furthermore, the fact that the severity of PTSD symptoms was mild to sub-clinical in the double diagnosis group suggests a significant contribution of general anxiety and depression symptoms in the expression of complaints in the PTSD and MTBI/PTSD groups.

### 4.2. Cumulative Effect of MTBI and PTSD (Question 2)

Due to the limited number of participants in the MTBI/PTSD group, data must be interpreted with caution. Nonetheless, some qualitative observations made in the present study could seem consistent with the suggestion that the comorbidity MTBI/PTSD could produce a cumulative detrimental effect on the expression of cognitive and behavioral symptoms. Again, more quantitative data in the future are necessary to confirm these hypotheses. Further, it is important to note that the specific contribution of PTSD symptoms to these complaints is even more difficult to establish in the MTBI/PTSD group, because the severity of PTSD symptoms was much less intense in this group than in the PTSD group, whereas the intensity of anxious and depressive symptoms between the two groups was comparable.

### 4.3. External Validation of Symptoms by Relatives (Question 3)

One of the original elements in the present study was that the complaints reported by the participants with PTSD (with or without MTBI) were subjected to external validation by relatives. The fact that only the MTBI/PTSD group’s complaints on the FrSBe dimensions were validated by relatives supports the hypothesis of a “cumulative effect” of MTBI and PTSD on “more visible” behavioral changes. In fact, despite PTSD participants’ reports of disinhibition, their relatives did not perceive a major change on this variable. However, relatives were more likely to identify apathy and disorganization in the PTSD group than in the control group.

Altogether, the results of the present study support the hypothesis that the presence of post-traumatic symptoms and of significant comorbidity can modulate reports of cognitive and behavioral symptoms in individuals with PTSD. The results further suggest that cognitive and behavioral problems could be exacerbated in individuals with more severe traumatic, depressive and anxious symptoms. However, these interpretations do not entirely explain the results obtained, particularly regarding the double diagnosis group.

### 4.4. Alternative Explanations

Anxious and depressive comorbidity in PTSD: Only the MTBI/PTSD group was validated by relatives on their reported difficulties in emotional self-control, apathy and executive dysfunction. This group also reported more forgetfulness than did the other clinical groups. However, it is difficult to attribute this result to the post-traumatic stress condition, since there is a comorbidity in PTSD groups (PTSD and MTBI/PTSD). Further, if post-traumatic symptoms have a major impact on participants’ reported complaints, we would expect the PTSD group to report at least as many problems as the double diagnosis group, particularly concerning irritability and forgetfulness, since trauma symptoms are more severe in the PTSD group than in the MTBI/PTSD group. These observations suggest that the greater reported complaints in the PTSD and MTBI/PTSD groups, as compared to the MTBI group, can be attributed at least in part to psychological comorbidity.

Litigation: One could argue that the greater number of complaints in the PTSD group may be attributable to involvement in a litigation process at the time of evaluation. However, it was not expected that the effects disappeared once the participants in the process of litigation were removed from the analyses, but rather that the results remained the same. This outcome suggests that the significant subjective adjournment of complaints by the groups is not artificially inflated by the inclusion of subjects that are in the process of litigation to make their difficulties recognized.

Elapsed time since the trauma: A third possible explanation for the results concerns the observed significant difference between elapsed time since the trauma in the PTSD group and MTBI/PTSD groups, respectively, at the time of evaluation. The latter group reported less elapsed time (average of nine months) than did the PTSD group (average of 48 months). This result may suggest the presence of a persistent adverse impact of MTBI injury in the double diagnosis group; these participants may have only recently begun facing the difficult post-trauma sequelae or may have less well-developed coping skills.

Interaction between emotional distress and MTBI sequelae: One plausible explanation for the greater reported disinhibition and forgetfulness in the MTBI/PTSD group is an interaction between significant emotional distress and a subtle decrease in self-regulation capacities, due to the damage to the fronto-temporal part of the brain during the traumatic event [1,18,19,20,21]. Over the past few years, neuroradiology data collected with functional imaging techniques have provided evidence of subtle neurological sequelae in the frontal and/or temporal lobes of the brain in certain subgroups of individuals with MTBI [20,22,23,24]. The subtle neurological damages may explain the presence of persistent post-concussive sequelae beyond the expected psychological reaction to PTSD in individuals with MTBI/PTSD [25,26].

### 4.5. Clinical and Research Considerations

The principal contribution of the present study was an increased understanding of the nature and severity of cognitive and behavioral complaints reported by individuals with PTSD and MTBI, taking comorbidity and antecedent factors into consideration. The results are partially compatible with findings from Hoge, McGurk [10] and Chamelian and Feinstein [8], which reported that post-traumatic and depressive symptoms caused memory and attention problems in individuals with PTSD post-trauma. From a clinical perspective, the current study presents some cues about the degree of congruence between participants’ and relatives’ perceptions of symptoms, as well as the extent of discrepancy in perception. Such observations could improve the comprehension of PTSD symptoms among patients and relatives alike, as well as facilitate the development of personalized interventions.

### 4.6. Strengths and Limitations of the Study

A major strength of the study was that participants’ medical antecedents were controlled. The inclusion of a control group, paired by age, education and gender with the clinical group, allowed us to document and to compare the frequency of cognitive and behavioral complaints in a normative sample. Instead of using only general questions about cognitive symptoms, the present study used three detailed self-report questionnaires to better capture the nature and intensity of cognitive and behavioral symptoms pre- and post-traumatic event. Diagnoses of MTBI and PTSD were established from medical history and through a formal interview based on DSM-IV-TR criteria [27]. The addition of inclusion and exclusion criteria allowed us to ensure that the reported symptoms were not attributable to pre-existing attention and learning deficits [28], alcohol abuse [29], brain injury [4] or medical conditions that could diminish cognitive capacities. The presence of psychological conditions commonly comorbid with PTSD and MTBI (e.g., anxiety, depressive symptoms and fatigue) were controlled.

One major limitation of the study is the size of the double diagnosis group. This small sample size limited the possibility of testing Vanderploeg *et al.*’s hypothesis [9] regarding a “cumulative” effect of concomitant MTBI and PTSD diagnoses on cognitive and behavioral complaints. A second significant limitation of the study was that the questionnaires used to measure cognitive and behavioral complaints have not been validated for PTSD and/or MTBI populations. This limitation restricts interpretation of the results and highlights the need to develop validated instruments specific to each clinical population.

### 4.7. Future Considerations and Clinical Implications

The results of the present study demonstrate the need for the further exploration of factors influencing the cognitive and behavioral changes perceived and reported by individuals with PTSD and MTBI and their relatives. The development of specific and sensitive instruments for each of these populations will allow finer evaluation of the overlap in cognitive and behavioral problems across neurological and psychiatric conditions. The cognitive and behavioral changes that develop following a traumatizing event often impact both the patients’ and family members’ lives; an improved comprehension of the changes that appear post-trauma would promote the development of personalized clinical interventions for individuals and for family members of individuals with PTSD and MTBI.

## 5. Conclusions

The present study was the first that was designed to directly compare subjective cognitive and behavioral complaints in PTSD (with and without MTBI) and MTBI (without PTSD) populations. The results confirmed that complaints vary not only by diagnosis, but also according to psychological condition. The fact that complaints reported in the PTSD group were partially noted by independent observers further validates the participants’ complaints. Finally, the results suggest that the presence of both MTBI and PTSD exacerbates psychological comorbidity and other symptoms, such as memory problems, at least during the first year after the trauma. Subtle fronto-temporal sequelae of MTBI, which may compromise self-regulation capacities and lead to deficits in cognitive and emotional regulation [18], have been proposed as alternative hypotheses to explain cognitive and adaptation problems in real life. In clinical settings where formal neuropsychological evaluation is not available and the use of self-report questionnaires for evaluating cognitive and behavioral symptoms may be recommended. The results of questionnaire measures could guide clinical interventions, such as psychoeducation and cognitive restructuring, to help PTSD victims and their family members better understand and cope with the cognitive and behavioral changes.
